# Editorial: Intelligent assistants for all

**DOI:** 10.3389/frobt.2026.1797990

**Published:** 2026-02-17

**Authors:** Nils Mandischer, Matthias Kraus, Junpei Zhong, Adriana Tapus

**Affiliations:** 1 Mechatronics, University of Augsburg, Augsburg, Germany; 2 Human-Centered Artificial Intelligence, University of Augsburg, Augsburg, Germany; 3 Department of Digital Innovation and Technology, Technological and Higher Education Institute of Hong Kong, Hong Kong, Hong Kong SAR, China; 4 ENSTA, Institut Polytechnique de Paris, Palaiseau, France

**Keywords:** assistive robotics, care robotics, elderly people, human-autonomy teaming, human-machine interaction, human-robot teaming, people with disabilities, social robotics

Demographic shifts have resulted in a growing population at risk of being excluded from fundamental aspects of daily life. Among them, the elderly and those with disabilities constitute some of the most marginalized members of our society. In response, political and academic discussions are increasingly emphasizing the potential of emerging technologies, such as robotics and AI, to address these challenges. This highlights the importance of collaborative efforts in researching and developing innovative methodologies and prototypes aimed at augmenting technological support to mitigate existing barriers. In the workplace, robotic and technological aids are essential for assisting with task completion, while in caregiving, social robots contribute to maintaining cognitive engagement among the elderly. Across various contexts, interactions between humans, robots, machines, and AI demonstrate significant potential for promoting inclusivity, ultimately advancing the goal of a fair and accessible society for all.

Including individuals with disabilities, whether stemming from age, congenital conditions, or injury, plays an important role in overcoming demographic challenges. However, its success depends on robust and accessible assistive technologies driven by novel methods for human-robot, human-machine, and human-AI interaction. This Research Topic explores innovative approaches to caregiving and workplaces that involve collaboration between individuals with disabilities and (partially) automated systems. However, the tendency towards tailored assistance risks perpetuating the perceived gap between individuals with disabilities and those without, underscoring the need for technical solutions that benefit all equally. These inclusive measures are necessary to achieve genuine equality and highlight the necessity for seamless support in caregiving and daily life.

In the Research Topic, we bring together interdisciplinary contributions that advance our understanding of how robotics and digital health technologies can augment human capacities, support wellbeing, and enable richer human-machine collaboration across emotional, cognitive, and physical domains. Salem and Sumi systematically induced embarrassment in human-robot interaction and showed that neutral and empathic robot responses, particularly when combined with an anime-style face, can meaningfully mitigate embarrassment by shaping emotional regulation and perceived social agency in interactions with a robot. These findings have important implications for designing emotionally intelligent robotic companions. Olatunji et al. employ a participatory design approach with the Stretch mobile manipulator, identifying the functional capabilities desired by older adults with cognitive and mobility impairments. They articulate key tasks, facilitators, and barriers for everyday robot support in home environments. Mitsugi et al. propose an AI-mediated framework rooted in experiential and organizational learning that transforms individual caregiver experiences into shared organizational knowledge to enhance engagement, reflection, and teamwork in elderly care settings. Schneider et al. introduce a graph-based model of communication for cooperative human-machine trajectory planning. The model reveals how closed communication loops beyond the action layer can enable consensual, emancipated cooperation, with simulation evidence showing multiple viable pathways to reach shared motion references. Finally, Reitelshöfer et al. present a marketplace-based architecture for socially adaptable robots that allows dynamic selection and adjustment of interaction characters, demonstrating feasibility and adaptability through core components such as scene analysis, agent bidding, and feedback.

Collectively, these contributions articulate a coherent vision of intelligent assistants that serve a diverse range of users, spanning applications from private homes and care environments to professional organization and shared environments. The works illustrate how intelligent assistants can be inclusive, context-sensitive, and scalable. By grounding technical innovation in human needs, lived experiences, and societal settings, this Research Topic underscores the potential of intelligent assistants to empower diverse users, support their autonomy and dignity, and meaningfully foster inclusion in daily life.

